# Amplification of miniature inverted-repeat transposable elements and the associated impact on gene regulation and alternative splicing in mulberry (*Morus notabilis*)

**DOI:** 10.1186/s13100-019-0169-0

**Published:** 2019-06-25

**Authors:** Youchao Xin, Bi Ma, Zhonghuai Xiang, Ningjia He

**Affiliations:** State Key Laboratory of Silkworm Genome Biology, Southwest University, Beibei, Chongqing, 400715 China

**Keywords:** MITEs, Amplification, Gene expression, Small RNA, Alternative splicing

## Abstract

**Background:**

Miniature inverted-repeat transposable elements (MITEs) are common in eukaryotic genomes, and are important for genomic evolution.

**Results:**

In the present study, the identification of MITEs in the mulberry genome revealed 286,122 MITE-related sequences, including 90,789 full-length elements. The amplification of mulberry MITEs and the influence of MITEs on the evolution of the mulberry genome were analyzed. The timing of MITE amplifications varied considerably among the various MITE families. Fifty-one MITE families have undergone a single round of amplification, while the other families developed from multiple amplifications. Most mulberry MITEs were inserted near genes and some could regulate gene expression through small RNAs. An analysis of transgenic plants indicated that MITE insertions can upregulate the expression of a target gene. Moreover, MITEs are frequently associated with alternative splicing events (exonizations).

**Conclusion:**

The data presented herein provide insights into the generation of MITEs as well as their impact on gene regulation and evolution in mulberry.

**Electronic supplementary material:**

The online version of this article (10.1186/s13100-019-0169-0) contains supplementary material, which is available to authorized users.

## Background

Miniature inverted-repeat transposable elements (MITEs) are very short, deletion derivatives of autonomous DNA transposons [1, 2]. They were originally discovered in the maize genome, and are widespread among animals and plants [3, 4]. MITEs and autonomous DNA transposons share common characteristics, including the presence of a terminal inverted-repeat (TIR) flanked by a target site duplication (TSD). The TIR and TSD suggest that most MITEs are derived from autonomous DNA elements, including *Tc1/Mariner* elements [5], *PIF/Harbinger* [1, 6, 7], *hAT* [8, 9], and *Mutator* [10]. Although MITEs lack an open reading frame encoding a transposase, their transposition is mediated by transposases associated with autonomous DNA transposons [11, 12].

In plants, MITEs often cover a considerable portion of the genome, including up to 10% of the *Oryza sativa* genome and 8% of the *Medicago truncatula* genome [13]. In rice, active MITEs have been detected (e.g., *mPing* and *mGing*) [11, 14], and the *Stowaway* MITE has been developed as a genetic engineering tool for transferring heterogeneous genes [15]. MITEs are often transcribed with plant genes [16, 17], and can influence genomic evolution and gene expression [18–22]. Specifically, MITEs usually downregulate gene expression [17, 23, 24], but MITEs containing regulatory motifs can have the opposite effect [20, 22]. Furthermore, MITEs can encode small RNAs that regulate the expression of target genes at the transcriptional or post-transcriptional levels [17, 25]. The structural similarity between MITEs and microRNA genes suggests that MITE-derived small RNAs may be generated via the microRNA pathway [26]. However, the MITE-derived small RNAs in Solanaceae species are most likely generated by the small interfering RNA (siRNA) biogenesis pathway [17]. Additionally, small RNAs derived from MITEs are important for silencing transposable elements (TEs) through stem-loop structures [27].

Alternative splicing is a common post-transcriptional regulatory process that increases transcriptome and proteome diversity in eukaryotic organisms. Alternative splicing is reportedly important for development [28, 29] and stress responses [30, 31]. Analyses of RNA sequences have revealed abundant alternatively spliced, intron-containing transcripts in *Arabidopsis thaliana* (61%), *Oryza sativa* (33%), *Zea mays* (40%), and *Glycine max* (63%) [32–35]. Moreover, alternative splicing based on TE activities has been confirmed in previous studies. For example, more than 5% of the alternative splicing occurring in humans is associated with *Alu* elements [36]. Additionally, in *A. thaliana*, more than half of the expressed *Ty1/Copia* elements are spliced [37]. Another study proved that *Alu* elements help modulate alternative splicing [38]. However, it remains unclear whether MITEs can mediate alternative splicing in plants.

Mulberry (*Morus* sp.) is a well-known food source for silkworms (*Bombyx mori* L.) and is an economically, ecologically, and medically important plant species. *Morus notabilis* has a relatively small genome (approximately 357 Mb), which has been sequenced [39]. Its genomic data may be useful for thoroughly characterizing mulberry MITEs. In this study, we identified the MITEs in the mulberry genome and analyzed their amplification patterns, effects on gene regulation, and evolution.

## Results

### Identification of MITEs in the mulberry genome

A total of 286,122 MITE-related sequences were detected in the mulberry genome, including 90,789 (31.73%) full-length elements (Table 1). The sequences of all MITE families and their distribution in mulberry genomes are available from the *Morus notabilis* transposable element database (http://morus.swu.edu.cn/mntedb/). The MITE-related sequences covered 13.83% of the mulberry genome, which was more than the corresponding coverage of the *O. sativa* genome (10% [13]). On the basis of sequence similarities, the mulberry MITEs were classified into 232 families. The TIR and TSD sequences were used to categorize the mulberry MITE families into the following four superfamilies: *Tc1/Mariner* (5.11%), *PIF/Harbinger* (4.67%), *Mutator* (0.44%), and *hAT* (0.97%) (Table 1). The ratio of full-length to partial MITEs in individual superfamilies varied from 13 to 60%, with only *Tc1/Mariner* having a value exceeding 50% (Table 1).

**Table 1:** Summary of the MITE superfamilies identified in *Morus notabilis*

Superfamily	Family Number	Total Elements	Full-length Number	Partial-length Number	Full-length/Partial-length (%)	Percentage of Genome (%)	TSD
*Tc1/Mariner*	89	116,427	43,474	72,953	59.59	5.11	TA
*PIF/Harbinger*	74	89,616	15,560	74,056	21.01	4.67	TWA
*hAT*	21	10,288	1570	8718	18.01	0.97	8 bp
*Mutator*	8	8130	921	7209	12.78	0.44	9 bp
*DTx*	40	61,661	29,264	32,397	90.33	2.63	
Total	232	286,122	90,789	195,333	46.48	13.83	

*DTx* represents an unknown superfamily

### Amplification of MITE families in the mulberry genome

**Fig. 1:**
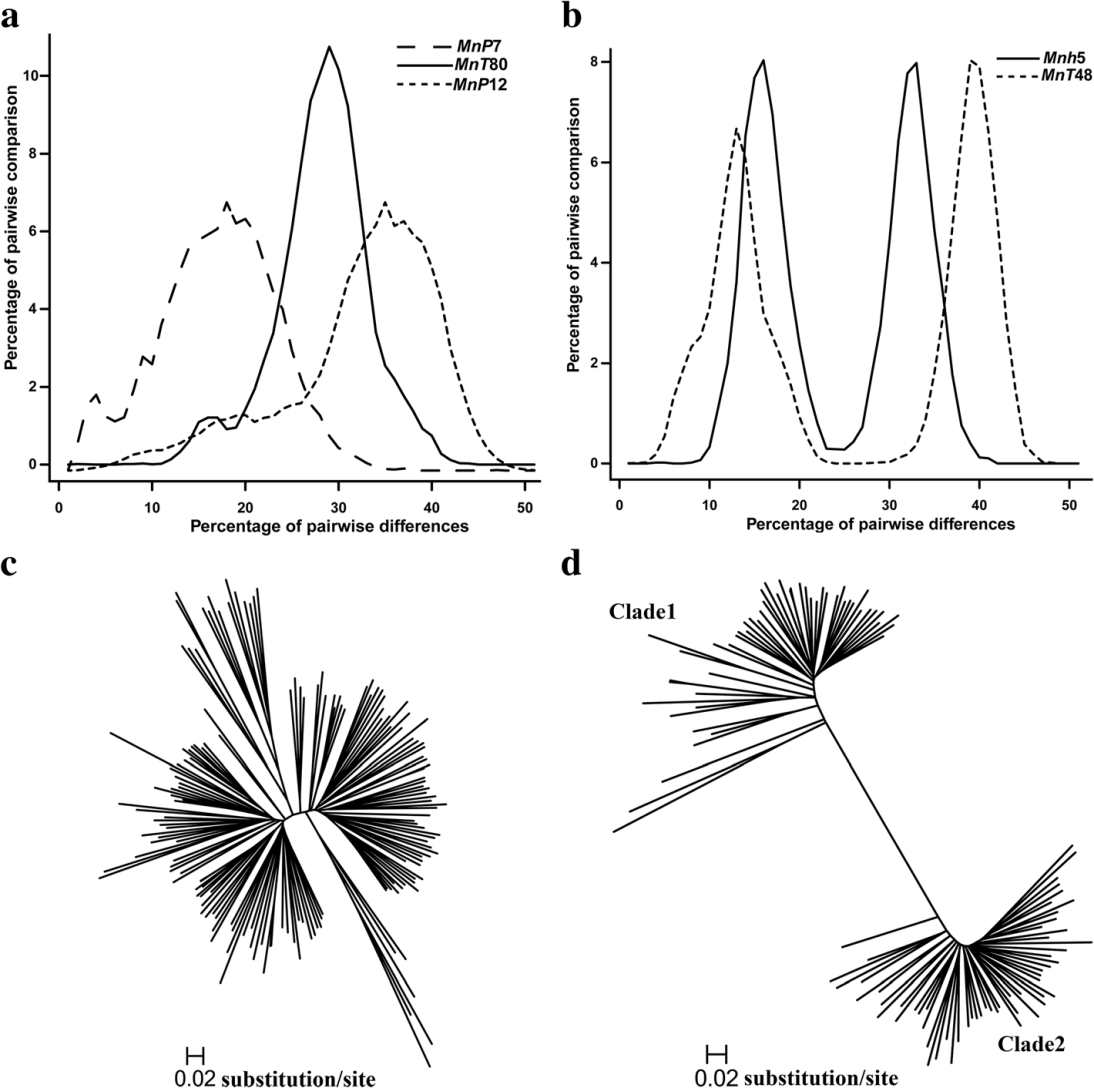
Rapid amplification of MITE families at different times. **a** Unimodal distribution of pairwise nucleotide diversity in some MITE families with full-length elements, implying only one amplification event occurred. Only three families are displayed. **b** Bimodal distribution, implying more than one amplification event occurred. Only two families are displayed. **c** Phylogenetic tree of the *MnT80* MITE family (with a unimodal distribution of pairwise nucleotide diversity). The star-shaped tree implies one amplification event occurred. **d** The phylogenetic tree of the *Mnh5* MITE family (with a bimodal distribution of pairwise nucleotide diversity) includes two well-supported clades

We investigated the amplification of 195 MITE families. Pairwise sequence diversities were calculated and histograms were drawn for the full-length MITE sequences from each family. A total of 100 families exhibited a multimodal distribution, while 51 and 44 exhibited unimodal and bimodal distributions, respectively (Fig. 1a and b). The wave histograms of the pairwise diversities suggested that each family underwent rapid amplification during evolution [40].

The histograms for these MITE families included unimodal peaks at different diversity levels, suggesting that the amplification of individual families occurred at distinct time points (Fig. 1a). The amplification time for *MnP7* (average pairwise nucleotide diversity of 0.160) was estimated to be 12.3 million years ago. In contrast, the amplification time for *MnP12* (average pairwise nucleotide diversity of 0.312) was estimated to be 24 million years ago [41].

Three phylogenetic trees were constructed for the families that produced unimodal and bimodal peaks. The MITE families with a unimodal peak distribution generated star-shaped phylogenetic trees, implying that their amplification was rapid and originated from a single master element (Fig. 1c). In contrast, the MITE families with a bimodal peak distribution were divided into two clades, implying that they had multiple ancestors or experienced multiple amplifications (Fig. 1d). Thus, some MITE families experienced one amplification event, while other families underwent multiple amplification events during evolution.

**Fig. 2:**
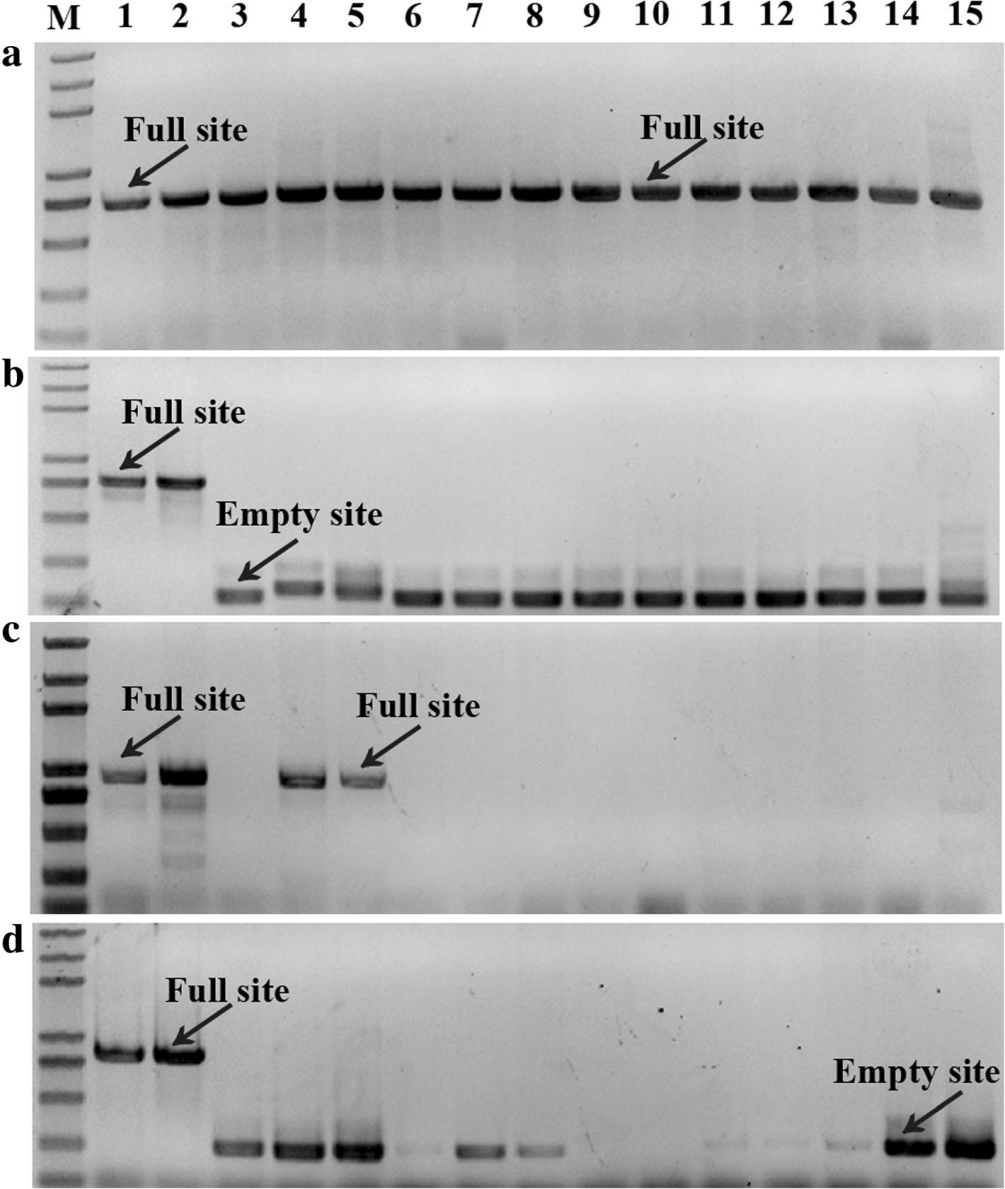
Site-specific PCR analysis. **a** *Mnh16*_scaffold1108_216257–216,737, **b** *Mnh16*_scaffold1960_162356–162,912, **c** *Mnh16*_scaffold93_462750–463,388, and **d** *Mnh16* _scaffold897_369175–369,745 in the following mulberry resources: 1, *M. notabilis*; 2, *M. yunnaneisis*; 3, *M. alba* var. Yun3; 4, *M. mongolica* Schneid.; 5, *M. wittiorum* Hand.-Mazz.; 6, *M. alba* var. Jinqiang63; 7, *M. alba* var. Taiwandaguo; 8, *M. alba* var. Xinjiaposijiguo; 9, *M. alba* var. Zhenzhubai; 10, *M. alba* var. Lunjiao109; 11, *M. alba* var. Multicaulis; 12, *M. alba* var. Hongguo1; 13, *M. alba* var. Hongguo2; 14, *M. alba* var. Da10; and 15, *M. nigra*. M, marker. Bands corresponding to a full site or empty site are indicated

**Fig. 3:**
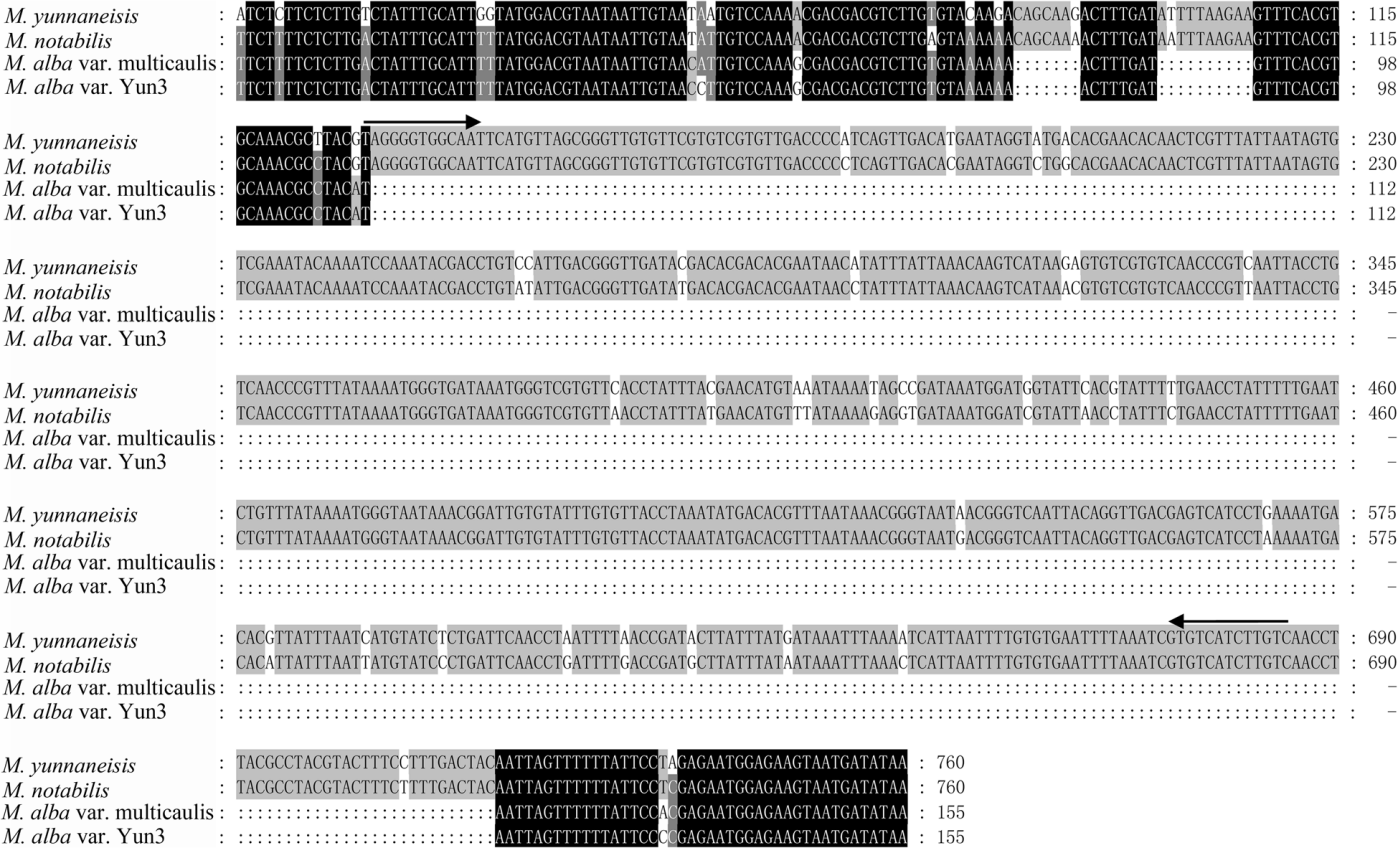
Multiple-sequence alignment of sequenced PCR bands (see Fig. 2b) for *M. yunnaneisis*, *M. notabilis*, *M. alba* var. Multicaulis, and *M. alba* var. Yun3. Arrows indicate the start and end points of the *Mnh16* sequence (557 bp). The flanking sequences are indicated on both sides of the arrow. *M. yunnaneisis* and *M. notabilis* contain the *Mnh16* MITE, while *M. alba* var. Multicaulis and *M. alba* var. Yun3 do not

To further explore MITE amplification, we studied the insertional polymorphism of MITEs in various mulberry species. Four polymorphic MITE loci were randomly chosen for PCR amplification using primers designed against the sequences flanking the MITEs (Fig. 2). The following three banding patterns were observed: (1) a band for the expected full site; (2) a band for the expected empty site; or (3) no band (i.e., no amplification). PCR amplification may have failed because the primers did not anneal to the expected sequence owing to a mutation in this region. We detected one locus that had MITEs in all analyzed samples (Fig. 2a and c), suggesting that this MITE insertion was fixed in *Morus* species. In some cases (Fig. 2b and d), the results clearly indicated that MITEs may have been activated following a polyploidization event. An analysis of the sequences of the extracted PCR bands for the expected full and empty sites confirmed that the difference between the sequences corresponding to the upper and lower bands in the gel was the presence or absence of a MITE (Fig. 3).

### Localization of MITEs in the mulberry genome

**Fig. 4:**
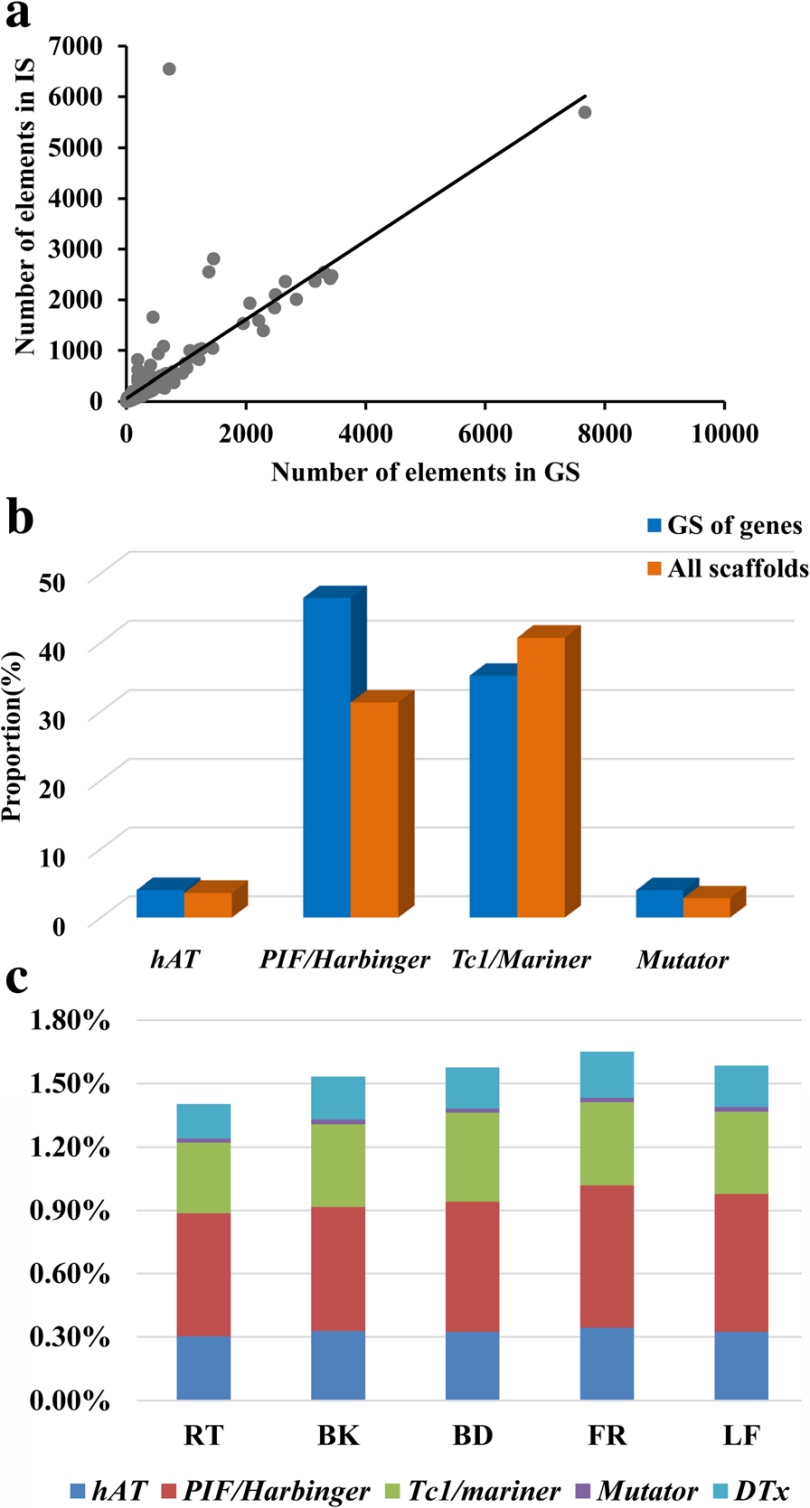
Distribution of MITEs in the mulberry genome. **a** Regression curve of the number of MITEs in GS and IS regions. Each MITE family was counted separately. **b** Analysis of the preferential distribution of each MITE superfamily in the 2000-bp sequences upstream of the start codon and downstream of the stop codon of genes. The ratios of the total number of each MITE superfamily to the total number of all MITEs in all scaffolds (orange) or in the 2000-bp sequences upstream of the start codon and downstream of the stop codon of genes (blue) are presented along the Y-axis. **c** Analysis of the expression of MITE-inserted genes. The ratios of the total number of expressed MITE-inserted genes to the total number of genes are presented along the Y-axis. Five tissues [root (RT), bark (BK), bud (BD), flower (FR), and leaf (LF)] were analyzed separately. Data for the four MITE superfamilies are presented in different colors

The MITEs inserted in gene sequence (GS) and intergenic sequence (IS) were counted, and the data were used to construct a regression curve for each family (Fig. 4a; *r*^2^ = 0.65, *P* < 0.05). Four MITE families had more elements in IS regions than expected (Fig. 4a), suggesting that these four MITE families were excluded from GS regions during evolution, while the other MITE families exhibited a linear and uniform distribution of elements. Moreover, for each superfamily, the distribution of MITEs in gene structure regions, as well as the 2000-bp sequences upstream of the start codon and downstream of the stop codon, were analyzed to evaluate whether the MITE insertion sites were preferentially close to genes (Fig. 4b). With the exception of the *Tc1/Mariner* superfamily, all MITE superfamilies tended to be inserted near genes.

After filtering out genes with a low RPKM value (i.e., < 1), the remaining genes with MITE insertion in exons were identified and analyzed. Among the five MITE superfamilies, the *PIF/Harbinger* MITEs had the highest expression ratio (i.e., the ratio of the total number of expressed MITEs to the total number of genes) (Fig. 4c). The proportions of MITE-associated genes that were expressed in five tissues were as follows: flower (1.65%), bud (1.58%), leaf (1.58%), bark (1.53%) and root (1.40%). These results implied that the MITE-associated gene expression ratios were relatively consistent among the five analyzed tissues.

### The *MnM2* MITE induced the ectopic expression of *MnANR*

**Fig. 5:**
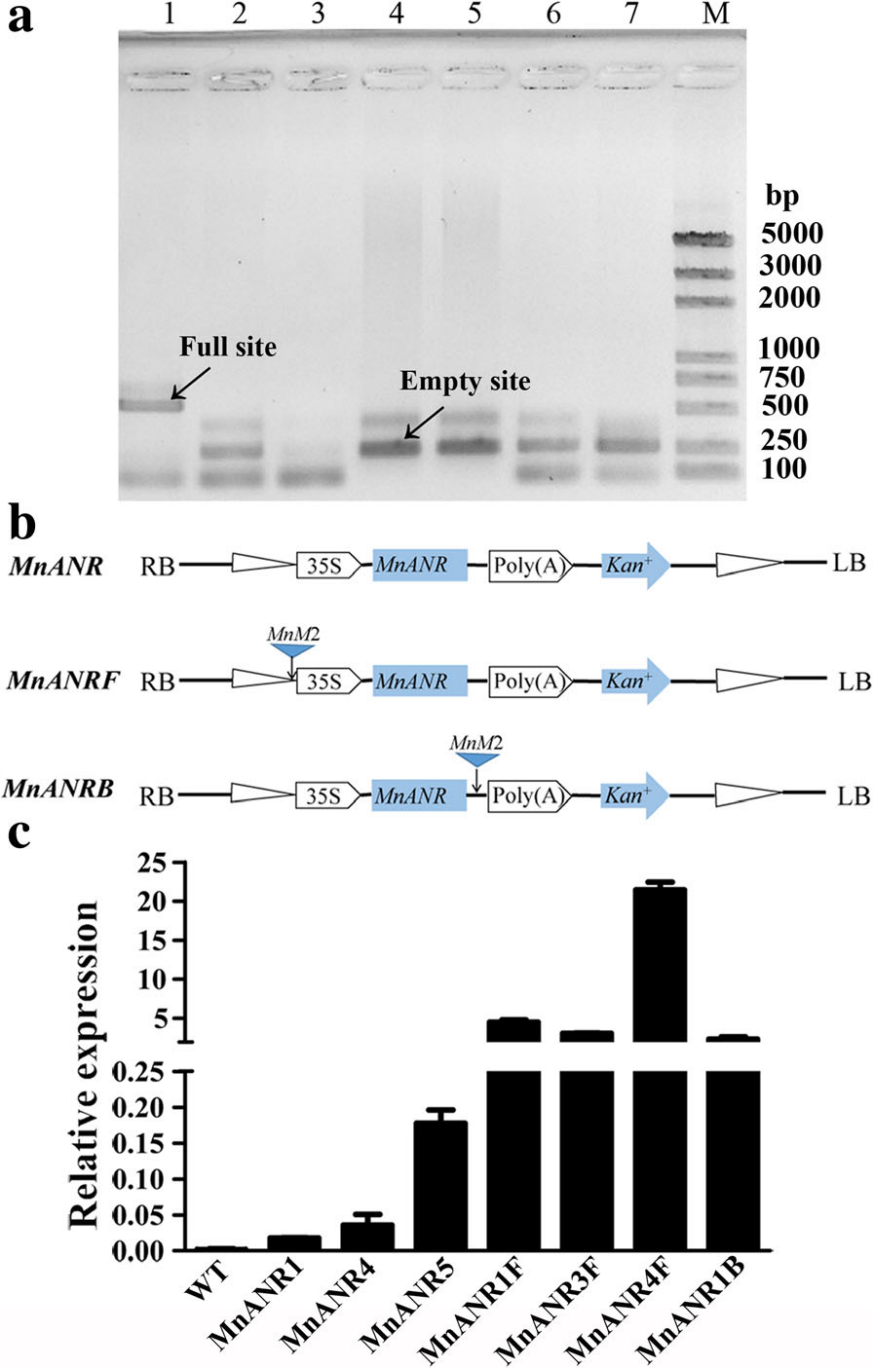
Effect of the *MnM2* element on *MnANR* expression in transgenic tobacco. **a** Site-specific PCR analysis using primers designed for sequences flanking *MnM2*_ scaffold96_203539–203,849 in the following mulberry resources: 1, *M. notabilis*; 2, *M. alba* var. Yun3; 3, *M. alba* var. Lunjiao109; 4, *M. alba* var. Jialing30; 5, *M. alba* var. Zhongsang5801; 6, *M. alba* var. Multicaulis; and 7, *M. nigra*. M, marker. Bands corresponding to the full site or empty site are indicated. **b** Schematic diagram of the *MnANR*, *MnANRMF*, and *MnANRMB* constructs. The *MnM2* sequence was inserted upstream of the *MnANRMF* construct and downstream of the *MnANRMB* construct. **c** Relative *MnANR* transcript levels in wild type (WT) and transgenic tobacco seedlings. *MnANR*, *MnANRMF*, and *MnANRMB* correspond to the constructs in **b**. Transcript levels are presented as fold changes relative to the tobacco actin gene. Error bars represent the standard deviation (*n* = 3)

In the current study, we examined the *MnM2* MITE inserted near the gene encoding the phosphate-responsive 1 family protein as well as the sequence polymorphisms in this region in different mulberry resources (Fig. 5a). The effect of *MnM2* on gene expression was subsequently investigated. Three expression vectors (Fig. 5b) were constructed and inserted into tobacco leaves via *A. tumefaciens*-mediated transformation. The expression levels of *MnANR*, which can change the color of tobacco flowers from red to white [42], in T_0_ transgenic tobacco seedlings were determined in a qRT-PCR assay. The expression levels in the transgenic tobacco seedlings were significantly higher than in the wild type. Additionally, transgenic seedlings carrying *MnM2* had significantly higher *MnANR* expression levels than those lacking *MnM2* (Fig. 5c). The activity of *MnANR* in the seedlings with *MnM2* was higher than that in the seedlings without *MnM2*, which led to a deeper change of tobacco color from red to white (see Additional file 2: Figure S1).

### Small RNAs derived from mulberry MITEs

**Fig. 6:**
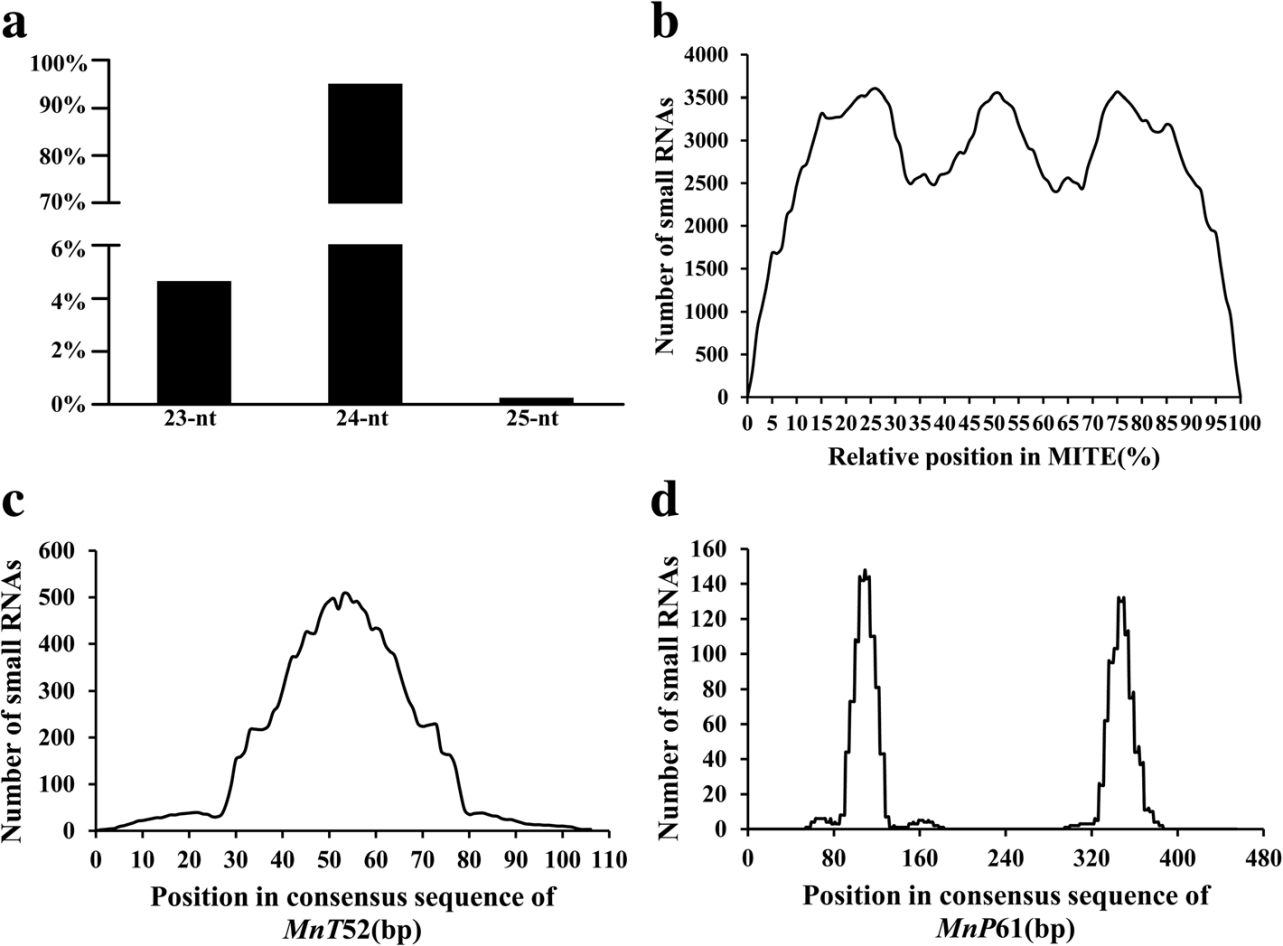
Length distributions and positions of MITE-derived small RNAs. **a** Length distribution of MITE-derived small RNAs. The ratios of the total number of small RNAs (for each length) to the total number of all small RNAs are presented along the Y-axis. **b** The relative positions of MITE-derived small RNAs in all full-length MITEs. **c** Small RNAs are predominantly generated from the central regions of MITEs in four of the 13 MITE families investigated. Only the *MnT52* family is presented. **d** Small RNAs are predominantly generated from the terminal regions of MITEs in six of the 13 MITE families investigated. Only the *MnP61* family is presented

In mulberry, 45,577 (15.9%) MITE sequences completely matched small RNA sequences. We analyzed the ratio of the number of small RNA-containing MITEs to the total number of MITEs in each superfamily. The proportions of MITEs in the four superfamilies were as follows: *PIF/Harbinger* (20.68%), *hAT* (20.13%), *Tc1/Mariner* (6.40%) and *Mutator* (3.43%) (Table 2). The *PIF/Harbinger* superfamily had the highest ratio of the number of small RNA-containing MITEs to the total number of MITEs. Additionally, there was no correlation between the ratio and the number of MITEs in each superfamily. Interestingly, 64.7% of the MITE-derived small RNAs were produced by MITEs located close to a gene (see Additional file 2: Figure S2). The mulberry MITE-derived small RNAs were 23–25 nt long, but 24-nt small RNAs were dominant (Fig. 6a). These observations were consistent with the reported results for Solanaceae species [17].

**Table 2:** Associations between MITEs and mulberry genes

Superfamily	Total Elements	Associated with Genes	Expressed with Genes	Related with small RNAs	Related with small RNAs/Total Elements (%)
*Tc1/Mariner*	116,427	34,418	743	7451	6.40
*PIF/Harbinger*	89,616	45,392	430	18,534	20.68
*hAT*	10,288	3924	204	2071	20.13
*Mutator*	8130	3897	13	279	3.43
Total	224,461	87,631	1390	28,335	12.62

The relative positions of small RNAs in MITEs were used to determine which part of the MITE sequences generated small RNAs because MITEs vary in length. For all MITE families, the parts of the full-length MITE sequences that could generate small RNAs were investigated. We observed that small RNAs with complete coverage were distributed throughout the MITEs, with one obvious peak in the central region and two at the termini (Fig. 6b). To investigate the variations in different MITE families, elements with nearly identical lengths from 13 MITE families were independently analyzed. Surprisingly, we detected considerable variations in the positions of small RNAs in different MITE families. Four of the 13 MITE families generated small RNAs mainly from the central region (Fig. 6c), while six other families generated small RNAs predominantly from the termini (Fig. 6d).

### Alternative splicing of mulberry genes related to MITEs

**Fig. 7:**
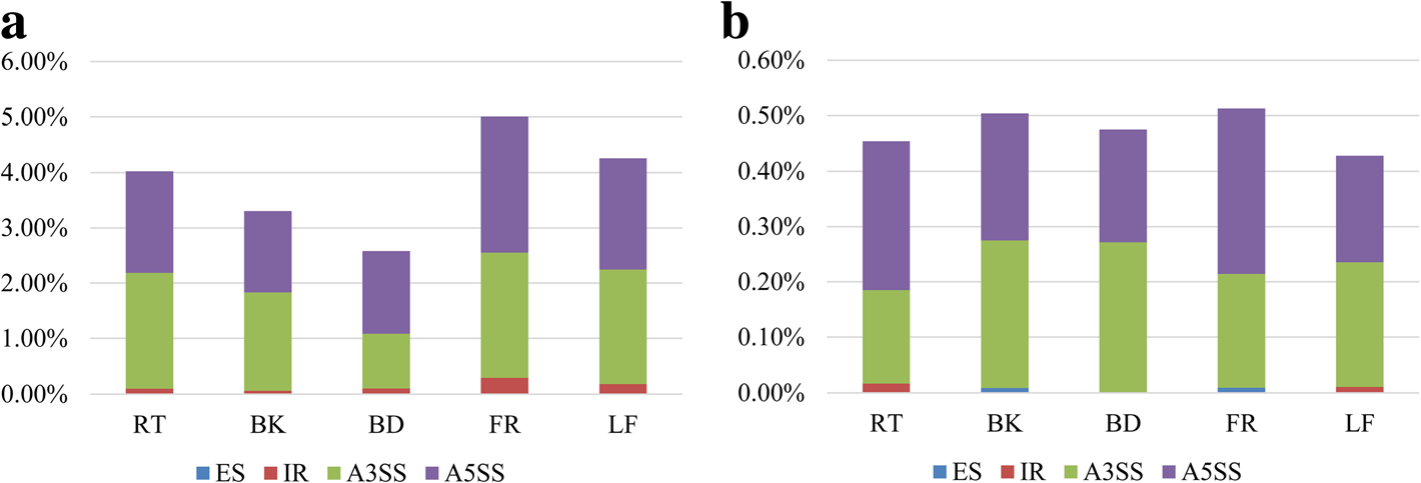
Analysis of alternatively spliced genes associated with MITEs. **a** The ratios of the total number of MITE-associated alternatively spliced genes to the total number of all alternatively spliced genes are presented along the Y-axis. **b** The ratios of the number of MITEs at alternative splicing sites to the number of all alternatively spliced genes are presented along the Y-axis. Five tissues [root (RT), bark (BK), bud (BD), flower (FR), and leaf (LF)] were analyzed separately. The four basic modes of alternative splicing [exon skipping (ES), intron retention (IR), alternative 3′ splice site (A3SS), and alternative 5′ splice site (A5SS)] are indicated in different colors

**Fig. 8:**
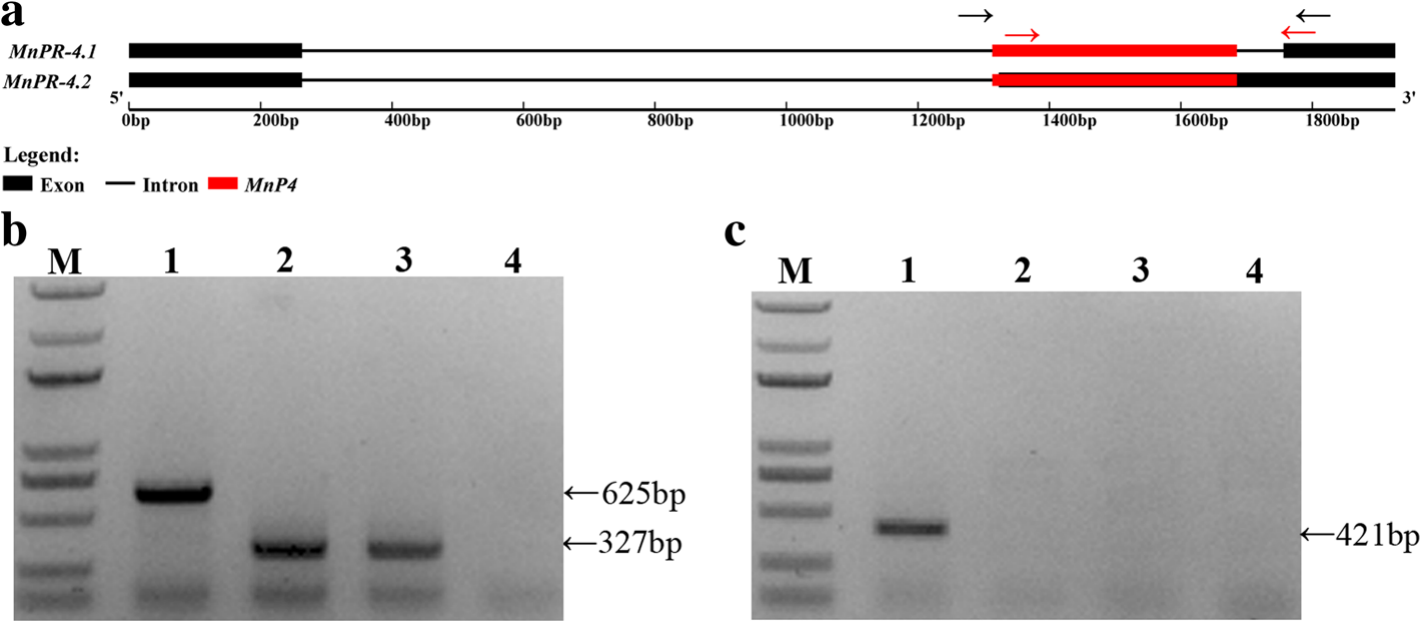
Alternative splicing of the *MnPR-4* gene related to MITEs in different mulberry resources. **a** Genomic structure of MITE-related *MnPR-4* splice variants. The arrowheads indicate primer positions for PCR amplification. **b** Site-specific PCR analysis using primers designed for the sequences flanking *MnP4*_scaffold205_783088–783,460. The primer positions are shown with the black arrows in **a**. **c** Detection of alternatively spliced transcripts. The reverse transcription PCR analysis of *MnPR-4* expression in the leaf. The primer positions are shown with red arrows in **a**. The PCR products were separated by agarose gel electrophoresis, and the resulting gel was stained with ethidium bromide. PCR product sizes are indicated on the right. 1–4 represent *M. notabilis*; *M. alba* var. Taiwandaguo; *M. alba* var. Hongguo1 and water. M, marker

Parts of MITEs can be retained in mature mRNAs via splicing (exonization), which is facilitated by sequence motifs that resemble splice sites (see Additional file 2: Figure S3 for a model of exonization). We comprehensively surveyed the association between MITEs and four basic modes of alternative splicing in five tissues. Of the four modes, alternative 5′ and 3′ splice sites were the predominant MITE-related modes (based on the ratio of the number of MITE-related alternative splicing events to the number of all alternative splicing events) (Fig. 7a). The proportion of MITE-related alternative splicing in five tissues was as follows: flower (5.00%), leaf (4.26%), root (4.02%), bark (3.31%) and bud (2.58%), (Fig. 7a); thus, MITEs had more exonization in flowers. Moreover, we analyzed the ratio of the number of MITEs at alternative splicing sites to the number of all alternative splicing events (Fig. 7b). The proportion of MITEs at alternative splicing sites in the five tissues was as follows: flower (0.51%), bark (0.50%), bud (0.48%), root (0.45%) and leaf (0.43%) (Fig. 7b), interestingly, the MITE ratio at the alternative splicing site was consistent across the five tissues compared to the MITE ratio at the exonization. To further verify the MITE-related alternative splicing, we analyzed a mulberry pathogenesis-related protein PR-4 gene (*MnPR-4*) in three mulberry resources (Fig. 8). The intron of this gene includes a MITE, which results in the second exon being spliced (Fig. 8a). Through PCR analysis, we found that *MnP4* was polymorphic in different mulberry resources (Fig. 8b). Through reverse transcription PCR analysis, we found that *MnP4* was missing from some mulberry resources, and that *MnPR-4* alternative splicing was also missing (Fig. 8c). These PCR products were verified by cloning and sequencing.

## Discussion

### Detection and characterization of mulberry MITEs

A previous study suggested that the number of MITEs is associated with genome size [13]. For example, papaya has a relatively small genome (342.68 Mb) and only one MITE family comprising 538 MITE-related sequences. Conversely, apple has a larger genome (881.28 Mb) and contains 180 MITE families with 237,302 MITE-related sequences [13]. In this study, however, we identified 232 MITE families with 286,122 MITE-related sequences in the *M. notabilis* genome (357 Mb) (Table 1). Although the mulberry genome is similar in size to that of papaya, it contains considerably more MITE sequences, likely because the mulberry MITEs underwent multiple amplifications during evolution. One amplification event was responsible for 26% of the MITEs in the mulberry genome. Additionally, *Tc1/Mariner* is the most abundant of the four known MITE superfamilies in mulberry, likely because of its proportion of full-length elements, which can be relatively easily activated.

### Intermittent generation of MITEs

The members of each MITE family are similar in sequence and length, which suggests the families underwent at least one amplification during evolution [43]. This suggestion was verified by histograms of pairwise nucleotide diversity among mulberry MITEs (Fig. 1). Analyses of different clades implied that many MITE families have undergone several rounds of amplification. The diversity in the amplification times among MITE families indicates these amplifications were not due to genome-wide duplications. It is possible that MITE amplifications in the *M. notabilis* genome occurred only sporadically. The MITEs may have been activated by a “genomic shock” or the temporary activation of a cognate transposase [44]. Indeed, in rice, *mPing* may have been activated by irradiation, cell culture, or domestication [11, 45, 46]. Most MITEs in the *M. notabilis* genome are relatively old and may be the result of selection or genetic drift during the long evolutionary history of the species.

### Impact of MITEs on mulberry gene expression

Our analysis suggested that *M. notabilis* MITEs are widely distributed in the genome and have been preferentially inserted upstream and downstream of genes (< 2000 bp), which is similar to what has been reported for other species such as *O. sativa* [47]. Although many MITEs are associated with genes, only a few studies have surveyed the effects of MITEs on gene expression [17, 20, 22–24]. In rice, the expression of the *Ghd2* gene is suppressed by a MITE in the 3′ untranslated region [24]. In maize, the variability at the *ZmNAC111* locus is likely caused by an 82-bp MITE inserted in the gene promoter, which may repress gene expression via the RNA-directed DNA methylation pathway [23]. In Solanaceae species, MITEs generate small RNAs and regulate gene expression through the small RNA silencing pathway [17]. A previous analysis of promoter activity revealed that MITE *kiddo* was responsible for approximately 20% of the expression levels of a neighboring gene in both transiently and stably transformed rice calli [22]. Moreover, when the methylation of *kiddo* was blocked with 5-azaC, the accumulation of *ubiquitin2* transcripts reportedly increased 3-fold [22]. These results indicate *kiddo* has dual functions that regulate gene expression. In this study, we detected transcriptional differences depending on the presence of MITEs in transgenic tobacco. Specifically, MITEs can influence the expression levels of genes with which they are associated. Thus, MITEs may have an effect on the evolution of gene expression. Furthermore, MITEs may be useful for regulating the expression of transgenes.

A thorough characterization of the impact of MITEs on mulberry gene expression requires a systemic investigation of MITE-derived small RNAs. The transcribed MITEs themselves may form double-stranded RNAs. Plant siRNAs include the 21-nt and 24-nt classes. A previous study determined that 21-nt siRNAs regulate mRNAs post-transcriptionally, while 24-nt siRNAs suppress gene expression at the transcriptional level via RNA-dependent DNA methylation and heterochromatin maintenance [48]. Our analyses uncovered 45,577 (15.9%) MITE sequences that completely matched a small RNA sequence. The MITE-derived small RNAs in the *M. notabilis* genome are mainly (95%) 24 nt long, similar to those in Solanaceae species [17]. Interestingly, 64.7% of the MITE-derived small RNAs were produced by MITEs located close to a gene, possibly because these MITEs may be more likely to be transcribed than those in intergenic regions. When considering all MITEs, small RNAs appear to be derived slightly more frequently from the central region and termini than from other regions (Fig. 6). However, the positions of small RNAs on MITEs vary considerably among the MITE families. The members of some MITE families produce small RNAs mainly from the central region, with very few from the termini, while the opposite trend occurs in other families. These differences are likely mediated by specific mechanisms that will need to be elucidated in future studies.

### The evolution of the mulberry genome was accelerated by MITEs

Our results revealed that many mulberry MITEs are located in alternative splice sites (Fig. 7). These observations imply that MITEs are important for alternative splicing. In mulberry, some MITEs are located in alternative splice sites and many MITEs are associated with alternative splicing. Moreover, most MITE-containing exons are alternatively spliced. This phenomenon suggests that alternative splicing caused by transposons may contribute to genetic disease. For example, an *Alu* insertion can reportedly cause a genetic disorder in humans [49, 50]. Transposable elements are a major contributor to the generation of lineage-specific exons in primates [51]. The insertion of TEs in intronic regions of genes may lead to mutations that create new exons [51, 52]. The exonization of TEs occurred frequently during human evolution. Moreover, noncoding RNAs, TEs, and alternative splicing are all involved in regulating gene expression. On the basis of our analyses of the mulberry genome, we determined that many noncoding RNAs are derived from MITEs. In human cells, 5S-OT regulates the *in trans* alternative splicing of multiple genes via TE/anti-TE pairings with target genes. MITEs can also form sense/antisense transcripts. We hypothesize that the insertion of MITEs in intronic regions of genes creates new exons, and such MITEs, some of which are alternatively spliced, were major contributors to the development of species-specific exons in mulberry. Additionally, these MITEs help modulate alternative splicing events. Furthermore, the exonization of MITEs can generate noncoding RNAs that may regulate gene expression levels. These processes may have enabled the evolution of mulberry species.

## Conclusions

Although many plant MITEs have been investigated, there have been few relevant studies on mulberry. We herein revealed the MITE amplification patterns in the mulberry genome. Our analyses of transgenic plants and small RNAs suggest that MITE insertions may regulate mulberry gene expression in diverse ways (e.g., epigenetic modifications and the production of new regulatory motifs). The frequent association of MITEs with alternative splicing and activation likely contributed to the evolution of mulberry. Our results provide insights into the generation of MITEs and as their contribution to the genetic regulation and evolution of mulberry.

## Methods

### Genome-wide identification of mulberry MITEs

Mulberry genomic sequences were downloaded from the MorusDB database (https://morus.swu.edu.cn/morusdb/). The MITE-Hunter, MITE Digger, and Repetitive Sequence with Precise Boundaries programs were used to identify potential MITEs in the mulberry genome with default parameters [53–55]. Candidate MITEs were detected based on the 80–80-80 rule to remove redundancies and were grouped into MITE families [56]. To mine all copies of each MITE family member, representative sequences with the expected length and structure for each family were used as queries to clarify the distribution and locations of MITEs in the mulberry genome with the RepeatMasker v3.2.9 program (http://www.repeatmasker.org/) and the Cross_match search engine.

All MITE families were classified into superfamilies based on similarities in the TSD and TIR sequences. Each MITE family was designated *MnX#*, where *Mn*, *X*, and *#* represent *M. notabilis*, the superfamily, and the family serial number, respectively. For the superfamily, *T*, *h*, *P*, *M*, and *N* correspond to *Tc1/Mariner*, *hAT*, *PIF/Harbinger*, *Mutator*, and unknown, respectively.

### Analysis of the amplification of MITE families

To assess the amplification of each MITE family, the MUSCLE v3.8 program was used to align the full-length sequences of the elements [57]. MITE sequences that were up to 3-bp short at their termini relative to the conserved element were considered to be full-length sequences. Neighbor-joining trees (pairwise deletion for gaps and the Kimura two-parameter substitution model) were constructed for each MITE family with the MEGA 6 program [58]. Pairwise nucleotide diversity was defined as the number of mismatches divided by the alignment length. Every gap was considered as a single mismatch. A Perl script was used to calculate and visualize the pairwise nucleotide diversity among MITE family members. A substitution rate of 1.3 × 10^− 8^ base substitutions per site per year was used to estimate the divergence time between two sequences [41].

### Survey of mulberry genomic variations caused by MITE insertions

All the primers used for PCR and qRT-PCR are listed in Additional file 1: Table S1. Genomic variations caused by MITE insertions were checked by PCR. Primers were designed based on the sequences flanking MITEs. The PCR amplifications were completed with DNA samples from the following 17 mulberry resources (*M. notabilis*, 2n = 2x = 14; *Morus yunnaneisis*, 2n = 2x = 14; *Morus alba* var. Yun3, 2n = 4x = 28; *Morus mongolica* Schneid., 2n = 5x = 35; *Morus wittiorum* Hand.-Mazz., 2n = 7x = 49; *M. alba* var. Jinqiang63, 2n = 4x = 28; *M. alba* var. Taiwandaguo, 2n = 4x = 28; *M. alba* var. Xinjiaposijiguo, 2n = 4x = 28; *M. alba* var. Zhenzhubai, 2n = 4x = 28; *M. alba* var. Lunjiao109, 2n = 4x = 28; *M. alba* var. Jialing30, 2n = 8x = 56; *M. alba* var. Zhongsang5801, 2n = 4x = 28; *M. alba* var. Multicaulis, 2n = 4x = 28; *M. alba* var. Hongguo1, 2n = 4x = 28; *M. alba* var. Hongguo2, 2n = 4x = 28; *M. alba* var. Da10, 2n = 5x = 35; and *Morus nigra*, 2n = 44x = 308). Each 20 μL reaction contained 20 ng genomic DNA, 2 mM PCR buffer, 0.2 mM each primer, 0.2 mM each dNTP, and 1 unit Taq polymerase with 2.5 mM MgCl_2_ (Takara Biotechnology Company, Dalian, China). The PCR amplification protocol was as follows: 94 °C for 4 min; 32 cycles of 94 °C for 30 s, 58 °C for 30 s, and 72 °C for 1 min; 72 °C for 7 min. The amplification products were sequenced and compared to further analyze MITE insertions.

### Mulberry genes associated with MITEs

Files with the coordinates of the predicted genes in scaffolds were obtained from the MorusDB database [59]. To determine whether MITEs were preferentially associated with predicted genes, all sequences were divided into two parts, namely the GS and IS. The GS included the region from 2000-bp sequences upstream of the start codon to 2000-bp downstream of the stop codon of genes (if the distance between two adjacent genes is less than 2000 bp, the spaced sequences were directly used in analyses). All other sequences were considered part of the IS. Additionally, a Perl script was written to identify MITE-associated genes. To test whether MITE superfamilies were preferentially associated with specific genomic regions, we determined the proportions of each superfamily in GS regions or in the scaffold.

The expression levels of MITE-inserted genes were analyzed in root, bark, bud, flower, and leaf tissues. Genes with MITEs inserted in their exons and an RPKM value (i.e., reads per kilobase of exon per million mapped reads) ≥ 1, were included in our analyses. The transcriptome data for the five analyzed tissues were downloaded from the MorusDB database.

### Vector construction, plant transformation, RNA isolation, and quantitative RT-PCR

The *M. notabilis* MITE sequences inserted near a gene were amplified by PCR using specific primers and then cloned into the *Bst*EII and *Hin*dIII or *Sal*I and *Eco*RI restriction enzyme sites of the pLGNL vector (upstream of the 35S promoter or downstream of the target gene). The target gene encoded an anthocyanidin reductase (*MnANR*, GenBank accession no. EXB31407.1), which can change the color of tobacco flowers from red to white [42], and was cloned into the *Sal*I and *Eco*RI or *Kpn*I and *Bam*HI restriction enzyme sites of the pLGNL vector. The correct orientation of the inserted amplified fragments was confirmed by DNA sequencing. The two recombinant vectors were introduced into *Agrobacterium tumefaciens* strain GV3101 cells, which were then co-cultivated with tobacco leaf sections as previously described [60].

Total RNA was isolated from the leaves of tobacco or mulberry plants with the TRIzol reagent (Invitrogen, USA). The extracted RNA was used as the template for synthesizing the first-strand cDNA with the PrimeScript™ RT-PCR Kit (Takara, Dalian, China). A quantitative real-time (qRT)-PCR assay was completed with the QuantiNova™ SYBR Green PCR Kit (Qiagen, Valencia, CA, USA) and the StepOnePlus™ Real-time PCR system (Applied Biosystems, USA). The *ACTIN* gene was used as an internal control for normalizing target gene expression levels.

### Identification of MITE-derived small RNAs

To identify MITE-derived small RNAs, mulberry leaf small RNA data were downloaded from the NCBI Sequence Read Archive database (project number: SRP032829; https://www.ncbi.nlm.nih.gov/sra/). All mulberry small RNA sequences were used as queries for BLAST searches for MITE sequences. Small RNA sequences that completely matched a MITE sequence were considered to be derived from the MITE [61]. These MITE-derived small RNAs were then mapped to the full-length MITE sequences to determine their positions in the MITEs. Because of the variability in MITE lengths, the small RNAs were mapped based on their relative positions in MITE sequences. For example, if a small RNA completely matched a 100-bp full-length MITE between nucleotides 30 and 50, the small RNA was mapped to the 30–50% region of this MITE. The number of small RNAs at each relative position was calculated and visualized. The number of small RNAs at each relative position was also investigated for individual MITE families. To limit biases, only families containing MITEs with similar lengths and more than 100 small RNAs were analyzed.

### Alternatively spliced mulberry genes associated with MITEs

Four basic modes of alternative splicing were analyzed (i.e., exon skipping, alternative 5′ splice site, alternative 3′ splice site, and intron retention). Alternatively spliced genes were considered to be associated with MITEs if the alternatively spliced sites were on MITEs or if MITEs were only found in alternative splicing variants of the genes. The analyses involved root, bark, bud, flower, and leaf tissues, and the transcriptome data for these five tissues were downloaded from the MorusDB database.

## Additional files

Additional file 1: **Table S1.** Primers used for PCR or qRT-PCR amplification of specific genes or transposable elements. (DOCX 14 kb)

Additional file 2: **Figure S1.** Tobacco flowers of wild-type (WT) and transgenic lines. Transgenic lines were transformed with *MnANR4*, *MnANR5*, *MnANR1F*, or *MnANR4F*. **Figure S2.** Analysis of the distribution of MITE-derived small RNAs in IS and GS regions. The ratios of the total number of MITE-derived small RNAs in IS or GS regions to the total number of MITE-derived small RNAs are presented along the Y-axis. **Figure S3.** Schematic model of MITE exonization. A MITE is inserted into the intron of a gene. During evolution, mutations within pseudo-splice sites activate the MITE insertion sites (marked by black arrows), and part of the MITE sequence is recognized as a new exon (‘exonized’). (ZIP 756 kb)

## Data Availability

Mulberry genomic and transcriptome sequences were downloaded from the MorusDB database. The mulberry leaf small RNA data were downloaded from the NCBI Sequence Read Archive database under the project number: SRP032829.

## Abbreviations

GS: Gene sequence
IS: Intergenic sequence
MITEs: Miniature inverted-repeat transposable elements
qRT-PCR: Quantitative real-time polymerase chain reaction
TIR: Terminal inverted-repeat
TSD: Target site duplication
