# Supplementary material for: Amplification of miniature inverted-repeat transposable elements and the associated impact on gene regulation and alternative splicing in mulberry (Morus notabilis)
Source: Mob DNA. 2019 Jun 25;10:27. doi: 10.1186/s13100-019-0169-0 (PMC6593561; doi:10.1186/s13100-019-0169-0)
Supplement: Supplementary file 1 — Table S1. Primers used for PCR or qRT-PCR amplification of specific genes or transposable elements. (DOCX 14 kb) [file 13100_2019_169_MOESM1_ESM.docx]

**Table S1** Primers used for PCR or qRT-PCR amplifications of specific genes or transposable elements.

| Gene/TE | Primer(5'→3') |
| --- | --- |
| *MnANR*-SalI for | GCGTCGACATGGCCACTCAGACCATC |
| *MnANR*-EcoRI rev | CGGAATTCTCAGATGTGAAGCAATCCT |
| *MnANR*-KpnI for | GGGGTACCATGGCCACTCAGACCATC |
| *MnANR*-BamHI rev | CGGGATCCTCAGATGTGAAGCAATCCT |
| *MnANR*-qRT for | TTATCAACTTGGGCTTGGAGG |
| *MnANR*-qRT rev | TCGGCTTCTGGTCGTTACATT |
| *NtActin*-qRT for | TCACAGAAGCTCCTCCTAATCCA |
| *NtActin*-qRT rev | GAGGGAAAGAACAGCCTGAATG |
| *Mnh16*_scaffold1108 for | TTTGTATTGCCGAGTTGTGTTT |
| *Mnh16*_scaffold1108 rev | AATTGCTAGCCCTATTGTGACG |
| *Mnh16*_scaffold1960 for | TTCTTTTCTCTTGACTATTTGC |
| *Mnh16*_scaffold1960 rev | TTATATCATTACTTCTCCATTC |
| *Mnh16*_scaffold93 for | CAAATACTCCTTAAAAGTGCATG |
| *Mnh16*_scaffold93 rev | TTGAATTGAAAATAACTTAAAAC |
| *Mnh16* _scaffold897 for | TTTAGTCCCGGAACTATAGGCTA |
| *Mnh16* _scaffold897 rev | CACTGATGAAAACTCGCAGAGAA |
| *MnM2*_ scaffold96 for | CGAATCAGGCAACGAATCTA |
| *MnM2*_ scaffold96 rev | AAAAGGGAAAAAAACACACC |
| *MnM2*-BstEII for | GGGTTACCTATTACGTCATATTGCTTATGTAGTACACT |
| *MnM2*-HindIII rev | CCAAGCTTGAGAAATTATTCAGTACACTAGGTGTATC |
| *MnM2*-SalI for | GCGTCGACTATTACGTCATATTGCTTATGTAGTACACT |
| *MnM2*-EcoRI rev | CGGAATTCGAGAAATTATTCAGTACACTAGGTGTATC |
| *MnPR-4-*splice for | TTTCTTATCGGTGTCAATC |
| *MnPR-4-*splice rev | ACTAAACGGGTCCTACTCT |
| *MnP4*_scaffold205 for | TAGCCCACCGTTGCTGCACTGAT |
| *MnP4*_scaffold205 rev | TTACATAGGACGCCCTCGGAAGA |
