# Supplementary figures and images for: Amplification of miniature inverted-repeat transposable elements and the associated impact on gene regulation and alternative splicing in mulberry (Morus notabilis)
Source: Mob DNA. 2019 Jun 25;10:27. doi: 10.1186/s13100-019-0169-0 (PMC6593561; doi:10.1186/s13100-019-0169-0)

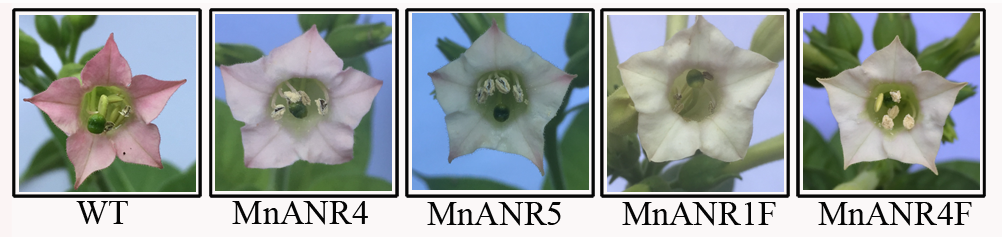

Supplement: Supplementary file 2 — Figure S1. Tobacco flowers of wild-type (WT) and transgenic lines. Transgenic lines were transformed with MnANR4, MnANR5, MnANR1F, or MnANR4F. Figure S2. Analysis of the distribution of MITE-derived small RNAs in IS and GS regions. The ratios of the total number of MITE-derived small RNAs in IS or GS regions to the total number of MITE-derived small RNAs are presented along the Y-axis. Figure S3. Schematic model of MITE exonization. A MITE is inserted into the intron of a gene. During evolution, mutations within pseudo-splice sites activate the MITE insertion sites (marked by black arrows), and part of the MITE sequence is recognized as a new exon (‘exonized’). (ZIP 756 kb) [file 13100_2019_169_MOESM2_ESM.zip › Fig S1.tif]

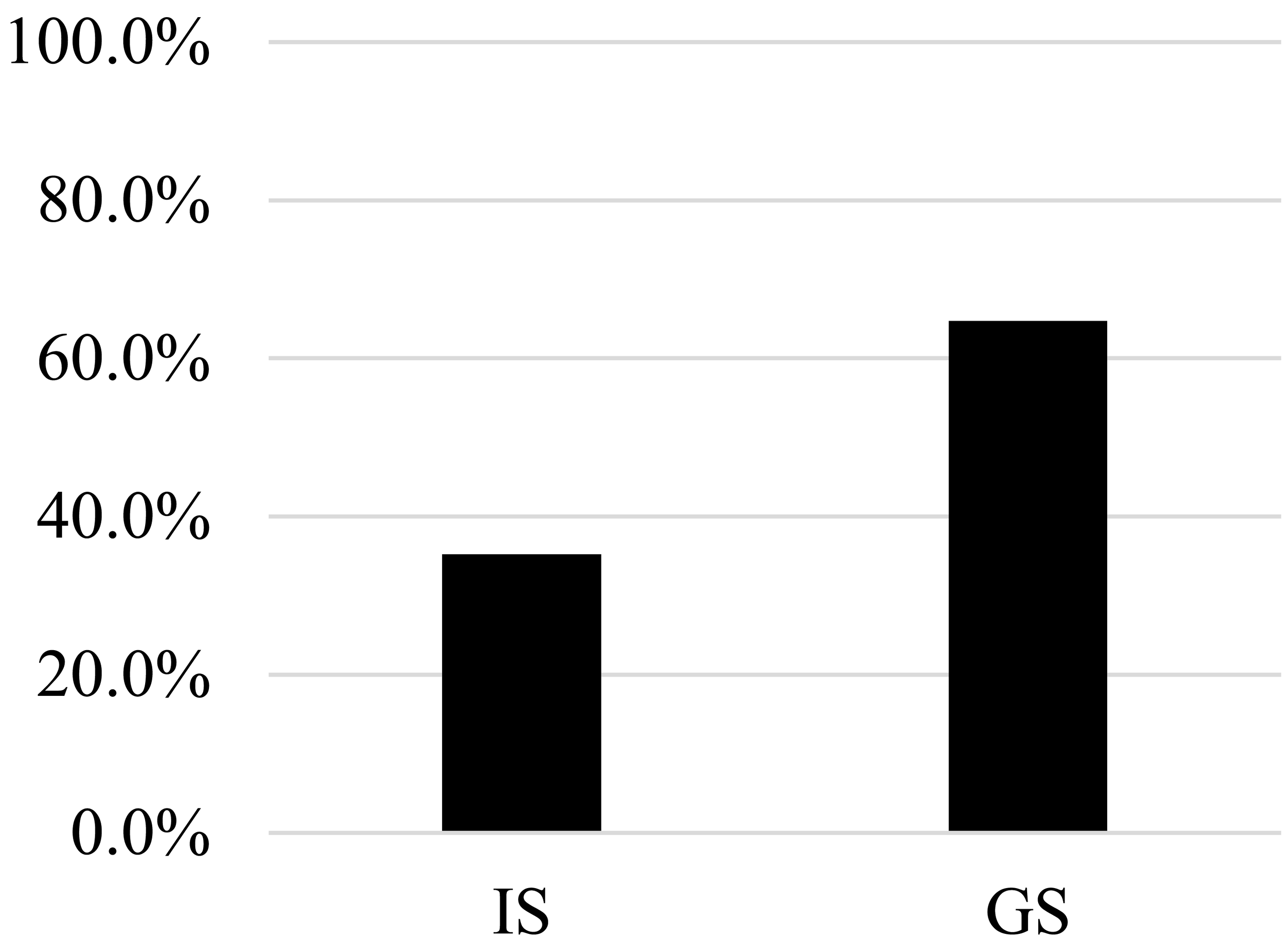

Supplement: Supplementary file 2 — Figure S1. Tobacco flowers of wild-type (WT) and transgenic lines. Transgenic lines were transformed with MnANR4, MnANR5, MnANR1F, or MnANR4F. Figure S2. Analysis of the distribution of MITE-derived small RNAs in IS and GS regions. The ratios of the total number of MITE-derived small RNAs in IS or GS regions to the total number of MITE-derived small RNAs are presented along the Y-axis. Figure S3. Schematic model of MITE exonization. A MITE is inserted into the intron of a gene. During evolution, mutations within pseudo-splice sites activate the MITE insertion sites (marked by black arrows), and part of the MITE sequence is recognized as a new exon (‘exonized’). (ZIP 756 kb) [file 13100_2019_169_MOESM2_ESM.zip › Fig S2.tif]

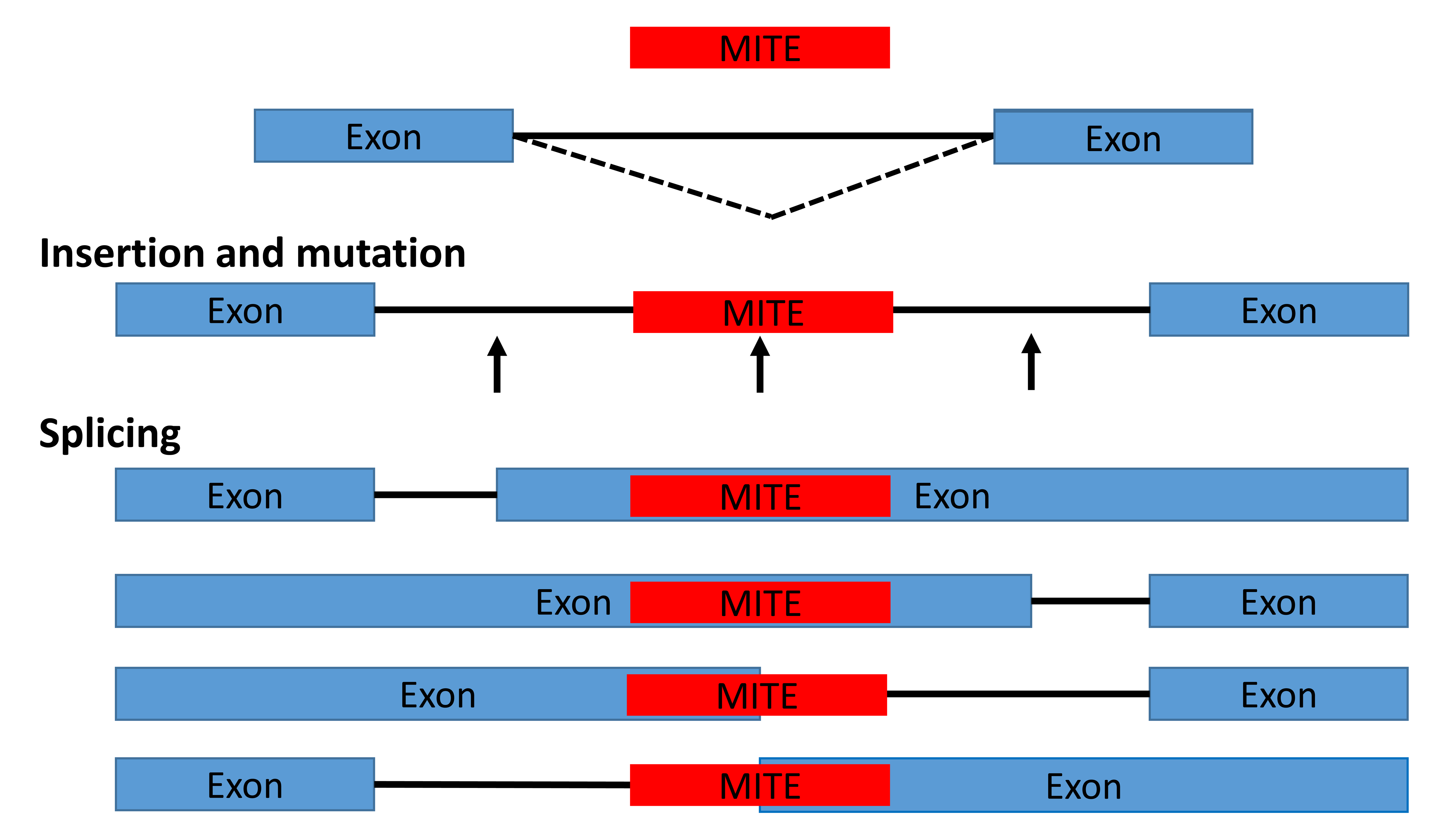

Supplement: Supplementary file 2 — Figure S1. Tobacco flowers of wild-type (WT) and transgenic lines. Transgenic lines were transformed with MnANR4, MnANR5, MnANR1F, or MnANR4F. Figure S2. Analysis of the distribution of MITE-derived small RNAs in IS and GS regions. The ratios of the total number of MITE-derived small RNAs in IS or GS regions to the total number of MITE-derived small RNAs are presented along the Y-axis. Figure S3. Schematic model of MITE exonization. A MITE is inserted into the intron of a gene. During evolution, mutations within pseudo-splice sites activate the MITE insertion sites (marked by black arrows), and part of the MITE sequence is recognized as a new exon (‘exonized’). (ZIP 756 kb) [file 13100_2019_169_MOESM2_ESM.zip › Fig S3.tif]
